# Down‐regulation of GAS5 ameliorates myocardial ischaemia/reperfusion injury via the miR‐335/ROCK1/AKT/GSK‐3β axis

**DOI:** 10.1111/jcmm.14724

**Published:** 2019-10-18

**Authors:** Nan Wu, Xiaowen Zhang, Yandong Bao, Hang Yu, Dalin Jia, Chunyan Ma

**Affiliations:** ^1^ The Central Laboratory of the First Affiliated Hospital of China Medical University Shenyang China; ^2^ Medical Research Center Shengjing Hospital of China Medical University Shenyang China; ^3^ Department of Cardiology The First Affiliated Hospital of China Medical University Shenyang China; ^4^ Department of Cardiovascular Ultrasound The First Affiliated Hospital of China Medical University Shenyang China

**Keywords:** growth arrest‐specific transcript 5, miR‐335, mitochondrial permeability transition pore, myocardial ischaemia/reperfusion injury, Rho‐associated protein kinase 1

## Abstract

Growth arrest‐specific transcript 5 (GAS5), along non‐coding RNA (LncRNA), is highly expressed in hypoxia/reoxygenation (H/R)‐cardiomyocytes and promotes H/R‐induced apoptosis. In this study, we determined whether down‐regulation of GAS5 ameliorates myocardial ischaemia/reperfusion (I/R) injury and further explored its mechanism. GAS5 expression in cardiomyocytes and rats was knockdown by transfected or injected with GAS5‐specific small interfering RNA or adeno‐associated virus delivering small hairpin RNAs, respectively. The effects of GAS5 knockdown on myocardial I/R injury were detected by CCK‐8, myocardial enzyme test, flow cytometry, TTC and terminal deoxynucleotidyl transferase dUTP nick end labelling (TUNEL) staining. qRT‐PCR and luciferase reporter assay were carried out to analyse the relationship between GAS5 and miR‐335. The regulation of GAS5 on Rho‐associated protein kinase 1 (ROCK1) expression, the activation of PI3K/AKT/GSK‐3β pathway and mitochondrial permeability transition pore (mPTP) opening was further evaluated. The results indicated that GAS5 knockdown enhanced the viability, decreased apoptosis and reduced the levels of lactate dehydrogenase and creatine kinase‐MB in H/R‐treatment cardiomyocytes. Meanwhile, down‐regulation of GAS5 limited myocardial infarct size and reduced apoptosis in I/R‐heart. GAS5 was found to bind to miR‐335 and displayed a reciprocal inhibition between them. Furthermore, GAS5 knockdown repressed ROCK1 expression, activated PI3K/AKT, thereby leading to inhibition of GSK‐3β and mPTP opening. These suppressions were abrogated by miR‐335 inhibitor treatment. Taken together, our results demonstrated that down‐regulation of GAS5 ameliorates myocardial I/R injury via the miR‐335/ROCK1/AKT/GSK‐3β axis. Our findings suggested that GAS5 may be a new therapeutic target for the prevention of myocardial I/R injury.

## 1 INTRODUCTION

Ischaemic heart disease is a leading disease with high mortality and disability rate in the world.^1^ The reestablishment of blood supply to ischaemic heart, also named myocardial reperfusion, is typically considered to be the best way to salvage the moribund myocardium to the utmost. However, the operation of myocardial reperfusion can itself cause further lethal myocardial damage, which is myocardial ischaemia/reperfusion (I/R) injury.^2^ Although the technique of myocardial reperfusion, such as primary percutaneous coronary intervention (PPCI), has been constantly improved, there is no effective way to prevent myocardial I/R injury.^3^

Long non‐coding RNAs (LncRNAs), a kind of RNA molecule typically over 200 nt in length and lack of protein‐coding activity, are implicated in pathogenic and pathophysiological processes.^4^, ^5^ Interestingly, emerging data from LncRNA array have showed that some LncRNAs are dysregulated in mice's myocardium during the process of I/R and suggested the potential regulatory roles of LncRNAs on myocardial I/R injury.^6^ Recently, growth arrest‐specific transcript 5 (GAS5), an LncRNA originally found in growth‐arrested cells,^7^ is found to be up‐regulated in hypoxia/reoxygenation (H/R)‐treated cardiomyocytes and promoted I/R‐induced myocardial apoptosis.^8^ Moreover, GAS5 is also found to be highly expressed in infarct boundary zone of acute myocardial infarct (AMI) rats and plays a regulatory role in the process of AMI.^9^ However, whether down‐regulation of GAS5 ameliorates myocardial I/R injury and its potential mechanism is still not determined.

Recent studies have demonstrated that LncRNA transcript sequences usually contain several microRNA (miRNA) binding sites,^10^ and LncRNA could regulate miRNA expression through specifically binding to miRNA, thereby regulating miRNA’ target (mRNA) expression, which is termed as an endogenous competitive RNA (ceRNA) regulatory network.^11^ Our previous study has demonstrated that miR‐335 plays an important role in myocardial I/R injury.^12^ Moreover, using a bioinformatics approach, GAS5 transcript was predicted to contain an miR‐335 binding region.^13^ Therefore, we speculated that GAS5 regulated myocardial I/R injury acting as a ceRNA for miR‐335.

In the present study, we determined the role of GAS5 in myocardial I/R injury and explored underlying mechanism in this process. We found that GAS5 knockdown ameliorated myocardial I/R injury in vitro and ex vivo. GAS5 was further demonstrated to regulate Rho‐associated protein kinase 1 (ROCK1) expression via binding to miR‐335, thereby modulating the PI3K/AKT/GSK‐3β signalling pathway and mitochondrial permeability transition pore (mPTP) opening.

## 2 MATERIALS AND METHODS

### 2.1 Cell culture

Rat adult cardiomyocytes (H9c2 cells) purchased from Shanghai Institutes for Biological Sciences and human adult ventricular cardiomyocytes (AC16 cells) obtained were, and the American Type Culture Collection were routinely maintained in DMEM mixed with 10% FBS, 100 units/mL penicillin and 100 μg/mL streptomycin in an incubator offering a 37°C with 5% CO_2_ condition.

Primary neonatal rat cardiomyocytes were isolated from 1‐day‐old Wistar rats and cultured as described previously. Briefly, neonatal rats were sacrificed by decapitation, and hearts were harvested. The ventricles were further separated and minced into 1 mm^3^ pieces, followed by digestion with 0.05% pancreatin and 0.01% collagenase. Then, cardiomyocytes were purified using differential attachment technique and cultured in Dulbecco's modified Eagle medium/F‐12 (DMEM/F12) containing 10% FBS, 100  U/mL penicillin and 100 μg/mL streptomycin. 5‐Bromo‐2‐deoxyuridine (Brdu) (0.1 mmol/L) was added to inhibit non‐cardiomyocytes proliferation.

### 2.2 Cell transfection

Small interfering RNA (siRNA) including GAS5‐siRNA and scrambled siRNA (negative control, NC) and microRNAs (miRNAs) including miR‐335 mimics, NC mimics, miR‐335 inhibitor and NC inhibitor which were designed and synthesized by GenePharma Co., Ltd. were transfected into cells using Lipofectamine 2000 (Invitrogen, Carlsbad, CA) following the manufacturer's instructions and recommendations. At 24 hours or 48 hours post‐transfection, cells were collected and processed for further analysis.

### 2.3 Hypoxia/reoxygenation model

H/R model was established to mimic myocardial I/R injury in vitro. Specifically, when the confluence of cardiomyocytes was 80%‐90%, the routine culture medium was replaced by Earle's medium without glucose and FBS, and cells were cultured in hypoxic condition (90% N_2_, 5% CO_2_ and 5% O_2_ in a tri‐gas incubator) at 37˚C for 6 hours. Afterwards, Earle's medium was removed, and the cells were cultured with routine medium in an incubator with 5% CO_2_ at 37˚C for 3 hours of reoxygenation.

### 2.4 Animals and gene therapy

A total of 24 healthy male Wistar rats, weighting 250 ± 10 g, obtained from Department of Laboratory Animal Science of China Medical University were divided into two groups (n = 12): (I) the rats were administrated by a single tail‐vein injection of 15 μL PBS diluting 5 × 10^10^ particles of adeno‐associated virus (AAV) delivering small hairpin RNAs (shRNAs) generated by Hanbio (Shanghai, China)per rat as previously described and^14^(II) the rats were given the same dose of scrambled shRNA(Shanghai, China) as control. Four weeks after the injection, the expression of GAS5 in left ventricular myocardium was determined using qRT‐PCR to examine the down‐regulation of GAS5. All treatment and use of animals in this study adhered to the Guide for the Care and Use of Laboratory Animals (NIH) and were authorized by the Institutional Animal Care and Use Committee of China Medical University.

### 2.5 Myocardial I/R injury in an isolated rat heart model

Rats were anesthetized by intravenous injection of pentobarbital sodium (100 mg/kg). Heparin (1500 IU/kg) was simultaneously given by an intravenous injection to prevent intracoronary thrombus formation. The rat heart was rapidly removed from the thoracic cavity into a 4°C heparinized Krebs‐Henseleit (K‐H) solution. Afterwards, the isolated heart was hung on a Langendorff perfusion device from the root of the aorta and perfused with 95% O_2_ + 5% CO_2_‐saturated K‐H solution under a constant pressure of 75 mm Hg at 37°C. The fluid‐filled latex balloon was inserted in the left ventricle via the left atrium and connected to a pressure transducer. Heart rate, left ventricular developed pressure (LVDP), positive first‐order derivative of ventricular pressure (+dp/dt) and negative first‐order derivative of ventricular pressure (−dp/dt) were recorded and analysed via a homodynamic system (MP150; BIOPAC Systems, Inc). All hearts underwent 30 minutes of ischaemia followed by 120 minutes of reperfusion as described in our previous research.^12^

### 2.6 qRT‐PCR

Total RNA was extracted from cardiomyocytes or left ventricular myocardium with TRIzol reagent (Invitrogen; Thermo Fisher Scientific, Inc). To detect GAS5 and ROCK1 mRNA expression, reverse transcription was performed using PrimeScript RT Reagent Kit with gDNA Eraser (TaKaRa) and quantitative PCR was performed using SYBR Premix Ex Taq II (TaKaRa) following to the manufacturer's protocols. To detect the amount of miR‐335 expression, reverse transcription was performed using Mir‐X™ miRNA First Strand Synthesis Kit (TaKaRa) and quantitative PCR was performed using Mir‐X™ miRNA qRT‐PCR SYBR^®^ Kit (TaKaRa) following to the manufacturer's protocols. β‐actin and U6 were used as an internal control. The primer sequences designed by Jinsirui Biotech Co., Ltd. were summarized in Table 1. The 2^−ΔΔCt^ method was carried out to analyse relative expression.^15^

**Table 1:** The information of primer sequences

Name	Sequences (5′ to 3′)
GAS5 forward (Rat)	TCTCACAGGCAGTTCTGTGG
GAS5 reverse (Rat)	ATCCATCCAGTCACCTCTGG
GAS5 forward (Human)	CTTCTGGGCTCAAGTGATCCT
GAS5 reverse (Human)	TTGTGCCATGAGACTCCATCAG
ROCK1 forward (Rat)	AGGCGGTGATGGCTATTATG
ROCK1 reverse (Rat)	TGTACGTCCCAACCAAAGAA
ROCK1 forward (Human)	AGGAAGGCGGACATATTAGTCCCT
ROCK1 reverse (Human)	AGACGATAGTTGGGTCCCGGC
miR‐335 forward (Rat/Human)	GCGGTCAAGAGCAATAACGAA
miR‐335 reverse (Rat/Human)	GTGCAGGGTCCGAGGTATTC

### 2.7 Cell Counting Kit‐8 assay

The cells were seeded into 96‐well plate (3000 cells per well), and the CCK‐8 assay (KeyGEN Biotech) was performed to detect cell viability following to the manufacturer's protocols.

### 2.8 myocardial enzyme test

When cardiomyocytes suffer from acute damage, lactate dehydrogenase (LDH) and creatine kinase‐MB (CK‐MB) will release into the culture medium. The activities of LDH and CK‐MB were detected using the LDH Assay Kit and CK‐MB Assay Kit (Jiancheng Biotech Co., Ltd) following to the manufacturer's instructions.

### 2.9 Apoptosis assay

To detect cell apoptosis in vitro, flow cytometry was carried out using the Annexin V‐Fluorescein Isothiocyanate (FITC)/Propidium Iodide (PI) Kit (Dojindo) following to the manufacturer's recommendations.

### 2.10 Myocardial submicroscopic structure observed by electron microscopy

Left ventricular myocardium was cut into the 1 × 1 × 1 mm size and fixed by glutaraldehyde stationary solution (2.5%) at room temperature for 24 hours and made into sections as previously described.^16^ The pathological changes in myocardial submicroscopic structure were observed under transmission electron microscopy.

### 2.11 Measurement of infarct size

The hearts were collected at the 120 minutes of reperfusion and frozen at −20°C. The freezing hearts were cut into 1 mm sections and incubated in 1% triphenyltetrazolium chloride (TTC) solution at 37°C for 20 minutes, followed by 4% paraformaldehyde fixation for 24 hours. The sections were photographed using a digital camera.

### 2.12 Terminal deoxynucleotidyl transferase dUTP nick end labelling assay

To detect apoptosis in myocardium, terminal deoxynucleotidyl transferase dUTP nick end labelling (TUNEL) assay was performed using In Situ Cell Death Detection kit following to the manufacturer's protocols. Each section was observed under a light microscope and photographed. Apoptotic cells were calculated in five random high magnified fields per section by two independent observers.

### 2.13 Luciferase activity assay

The AC16 cells prepared in 24‐well plate were cotransfected with wide type or mutant luciferase reporter gene carrier (pmirGLO‐GAS5‐wtor pmirGLO‐GAS5‐mut), which were generated by GenePharma Co., Ltd. along with miR‐335 mimics or NC mimics following to the manufacturer's protocols. After 48 hours transfection, luciferase activity analysis was carried out using the luciferase reporter system (Promega).

### 2.14 Sensitivity of mPTP to calcium ion

The sensitivity of mPTP to calcium ion (Ca^2+^) could reflect the opening of mPTP.^17^ To be specific_,_ the mitochondria in transfected cardiomyocytes were extracted from the cell lysates using Cell Mitochondria Isolation Kit (Beyotime) following to the manufacturer's recommendations. The reaction of mPTP to Ca^2+^ was detected using Purified Mitochondrial Membrane Pore Channel Colorimetric Assay kit (GENMED) following to the manufacturer's recommendations.

### 2.15 Immunohistochemistry analysis

Left ventricular myocardium was prepared into sections as previously described.^12^ The sections were submerged in 0.01 mol/L citrate buffer (pH = 6.0), followed by antigen retrieval with microwaves treatment. Then, these sections were in turn treated with 3% H_2_O_2_ and 10% goat serum to inhibit endogenous peroxidase activity and block non‐specific reactions. Afterwards, they were successively incubated with anti‐cleaved caspase‐3 (1:200; Abcam, Cambridge, UK) primary antibody overnight at 4°C and an HRP‐conjugated secondary antibody for 1 hours at room temperature, subsequently staining with the 3,3‐diaminobenzidine (DAB) solution (Santa Cruz Biotechnology) and counterstaining with haematoxylin.

### 2.16 Western blotting analysis

Proteins were extracted from cell lysates using RIPA lysis Buffer, and the protein concentration was measured using the Enhanced BCA Protein Assay Kit (Beyotime) following to the manufacturer's recommendations. Proteins were denatured by heat and then separated by SDS‐PAGE electrophoresis, finally transferred to PVDF membranes. The membranes were blocked using 1% bovine serum albumin solution for 1 hour at room temperature and then incubated with primary antibodies, including anti‐cleaved caspase‐3 (1:1000; Abcam), anti‐ROCK1 (1:1000; Abcam), anti‐phospho‐PTEN (1:1000; Abcam), anti‐PTEN (1:1000; Abcam), anti‐phospho‐AKT (1:1000; Abcam), anti‐AKT (1:1000; Abcam), anti‐phospho‐GSK‐3β (1:1000; Abcam), anti‐GSK‐3β (1:1000; Abcam) and anti‐β‐actin (1:1000; Zhongshan Jinqiao Biotechnology) at 4°C overnight, followed by incubation with HRP‐conjugated secondary antibody (1:5000; Zhongshan Jinqiao Biotechnology) at room temperature for 30 minutes. Protein bands were detected using a Immun‐Star HRPKit (Bio‐Rad) following to the manufacturer's protocols. Relative densitometry was analysed using Image J2x analysis software (NIH).

### 2.17 Statistical analysis

Data were expressed as the mean ± standard deviation (SD). To analyse the differences between two groups, Student's *t* test was conducted, and if analysing the differences between multiple groups, one‐way analysis of variance was used. *P* < .05 indicated a statistically significant difference. All statistical analysis was carried out using SPSS version 17.0 software (SPSS Inc).

## 3 RESULTS

### 3.1 GAS5 was up‐regulated in H/R‐cardiomyocytes and I/R‐myocardium

**Figure 1:**
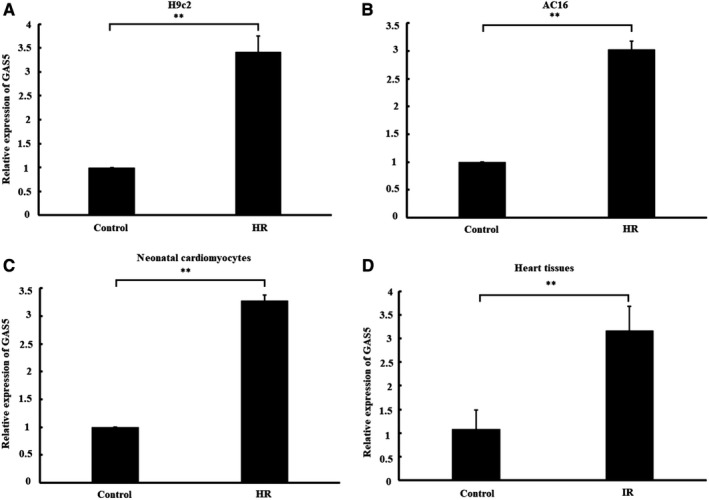
Expression of GAS5 in hypoxia/reoxygenation (H/R)‐cardiomyocytes and ischaemia/reperfusion (I/R)‐heart tissues. H9c2 (A), AC16 (B) and primary neonatal cardiomyocytes (C) were subjected to 6 h of hypoxia, followed by 3 h of reoxygenation (H/R). Cells underwent 9 h of normoxia as a control. Relative expression of GAS5 in cardiomyocytes was determined by qRT‐PCR. Data are expressed as the mean ± standard deviation (SD), n = 3, **P < .01. D, The isolated rat hearts were subjected to 30 min of global ischaemia, followed by 120 min of reperfusion (I/R). The isolated rat hearts were continuously perfused with K‐H solution without ischaemia as a control. Relative expression of GAS5 in heart tissues was determined by RT‐qPCR (n = 6 per group). ** *P* < .01

To explore the change in the expressed pattern of GAS5 in myocardial I/R injury, we detect the level of GAS5 in H/R‐cardiomyocytes and I/R‐myocardium using qRT‐PCR. As shown in Figure 1A and 1, GAS5 expression was remarkably increased in H/R‐treated H9c2, AC16 and primary neonatal cardiomyocytes compared with normoxic cardiomyocytes (*P* < .01). Similarly, we also found that GAS5 was highly expressed in I/R‐treated myocardium (Figure 1C) compared with normal myocardium (*P* < .01). The results obtained suggested that GAS5 expression was induced in heart by hypoxia or ischaemia insult.

### 3.2 Down‐regulation of GAS5 alleviated H/R‐induced cardiomyocyte injury in vitro

**Figure 2:**
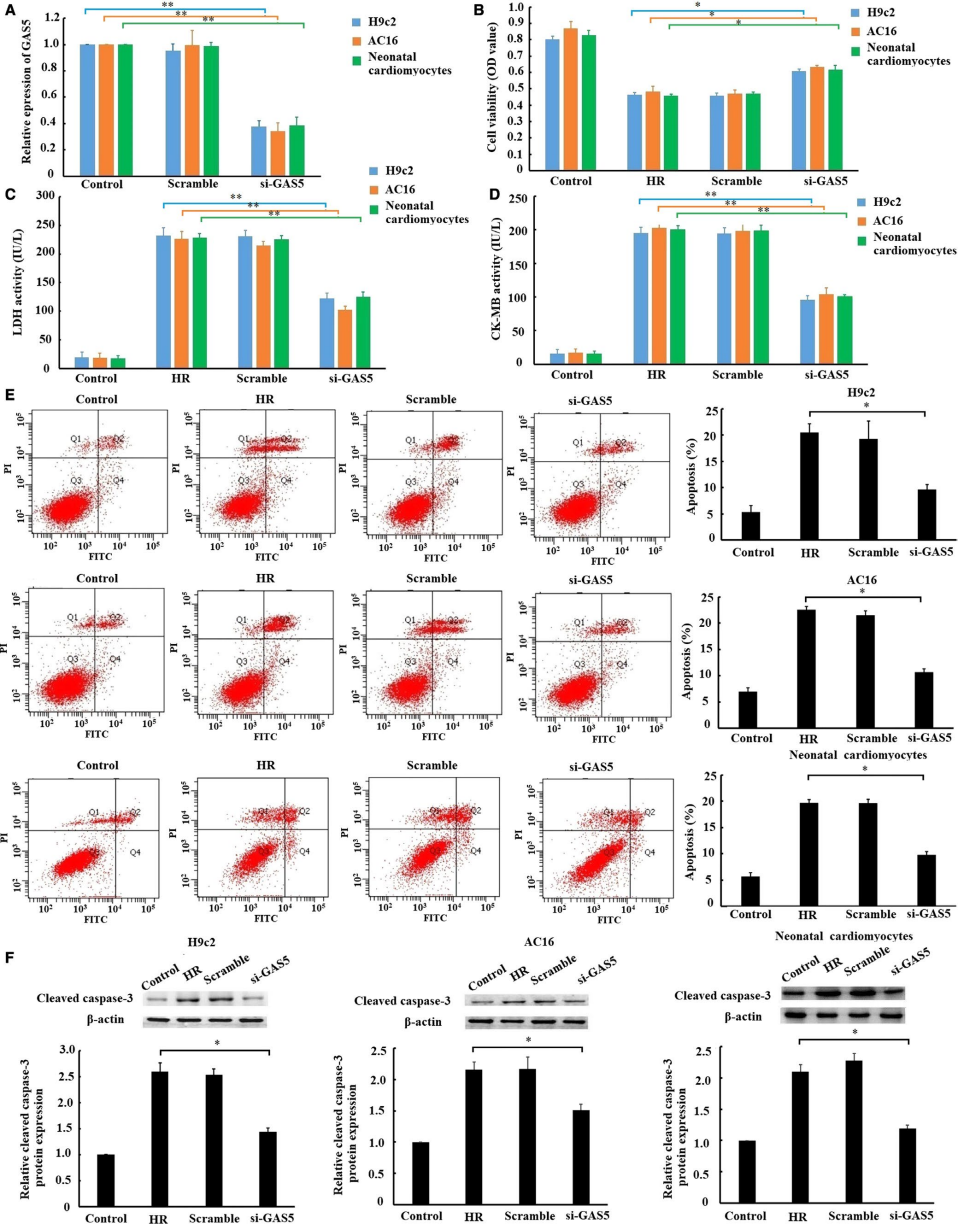
Effect of GAS5 knockdown on hypoxia/reoxygenation (H/R)‐induced cardiomyocyte injury in vitro. A, The expression of GAS5 in cardiomyocytes was measured by qRT‐PCR after transfected with GAS5‐specific small interfering RNA (siRNA), negative control (scramble). After 24 h of transfection, cells were subjected to 6 h of hypoxia, followed by 3 h of reoxygenation. Then, cell viability was measured by CCK‐8 assay (B). The lactate dehydrogenase (LDH) (C) and creatine kinase‐MB (CK‐MB) (D) activity in culture medium were measured by spectrophotometry. E, Cell apoptosis was detected by flow cytometry. F, Cleaved caspase‐3 protein expression was detected by Western blotting. Data are presented as the mean ± standard deviation, n = 3,* *P* < .05, ** *P* < .01

To explore the role of GAS5 in H/R‐induced cardiomyocyte injury, we observed the effects of GAS5 knockdown on the cell viability, release of cardiac enzymes and apoptosis in vitro. As shown in Figure 2A, GAS5 expression in H9c2, AC16 and primary neonatal cardiomyocytes was knockdown by transfected with si‐GAS5. Moreover, the cell viability was enhanced (Figure 2B), and the release of LDH (Figure 2C) and CK‐MB (Figure 2D) was reduced by transfected with si‐GAS5. As to apoptosis, flow cytometry and Western blotting showed a lower apoptosis rate (Figure 2E) and active caspase‐3 express (Figure 2F) in cardiomyocytes respectively by transfected with si‐GAS5. The results obtained suggested that down‐regulation of GAS5 alleviated myocardial I/R injury in vitro.

### 3.3 Down‐regulation of GAS5 ameliorated myocardial I/R injury in isolated rat hearts

**Figure 3:**
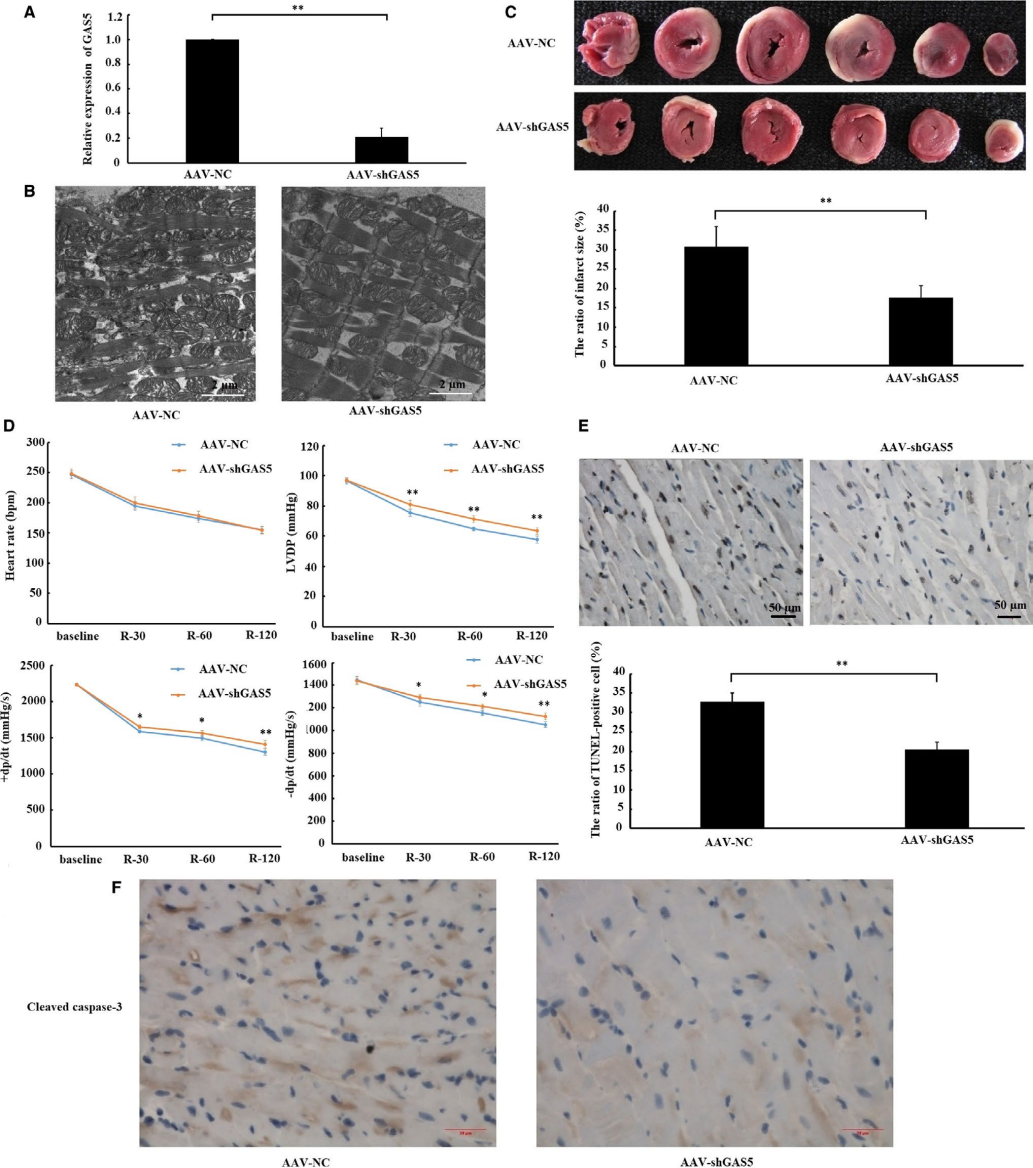
Down‐regulation of GAS5 alleviated I/R‐induced myocardial injury ex vivo. A, Rats were given a single tail‐vein injection of adeno‐associated virus carrying small hairpin RNAs (AAV‐shGAS5) and negative control (AAV‐NC). Four weeks after the injection, the expression of GAS5 in left ventricular myocardium was determined using qRT‐PCR. B, Myocardial submicroscopic structure was observed by transmission electron microscope (×5000). C, The effect of GAS5 knockdown on myocardial infarct size measured by TTC staining (n = 6 per group). The viable myocardium was stained red, and infarct myocardium was unstained. D,The effect of GAS5 knockdown on the changes of heart rate, left ventricular developed pressure (LVDP), positive first‐order derivative of ventricular pressure (+dp/dt) and negative first‐order derivative of ventricular pressure (−dp/dt). Baseline: cardiac function parameters were recorded 1 min before ischaemia. R‐30, R‐60 and R‐120: cardiac function parameters were collected after 30, 60 and 120 min of reperfusion (n = 6 per group). E, The effect of GAS5 knockdown on apoptosis measured by TUNEL staining (×400) (n = 6 per group). The apoptotic cell nuclei were stained brown, and the living cell nuclei were stained blue. F, The effect of GAS5 knockdown on cleaved caspase‐3 protein expression measured by immunohistochemistry (IHC) analysis (×400) (n = 6 per group). Data are expressed as the mean ± standard deviation (SD). **P < .05

To further determine the role of GAS5 on I/R‐heart, we observed the effects of GAS5 knockdown on the changes in myocardial submicroscopic structure, infarct size, cardiac function and apoptosis. As shown in Figure 3A, GAS5 expression in myocardium was significantly reduced by transduced with AAV‐GAS5shRNA. The swelling of the mitochondria was reduced and mitochondrial cristae were denser inAAV‐GAS5shRNA‐treated rats, as observed by electron microscopy (Figure 3B). Moreover, fewer myocardial infarct sizes were shown in AAV‐GAS5shRNA‐treated rats (Figure 3C). Meanwhile, cardiac parameters (LVDP and ± dp/dt) also indicated that cardiac function in AAV‐GAS5shRNA‐treated rats recovered better than that in AAV‐NC‐treated rats (Figure 3D). TUNEL staining and Immunohistochemistry (IHC) analysis also indicated lower apoptosis rate (Figure 3E) and active caspase‐3 express (Figure 3F) in AAV‐GAS5shRNA‐treated rat hearts, respectively. The results obtained suggested that down‐regulation of GAS5 ameliorated myocardial I/R injury ex vivo.

### 3.4 Interaction between GAS5 and miR‐335

**Figure 4:**
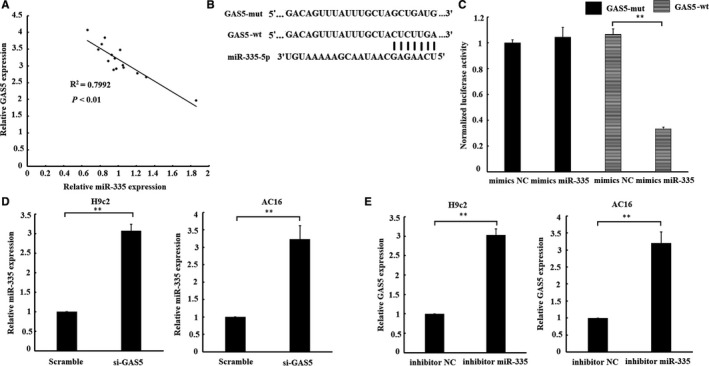
Interaction between GAS5 and miR‐335. A, a negative relationship between GAS5 and miR‐335 expression existed in ischaemia/reperfusion (IR)‐heart tissues. B, miRNA binding sites in the GAS5 sequence. C, Luciferase reporter gene assay validated the interaction between GAS5 and miR‐335 in AC16 cells by cotransfection with luciferase vector of GAS5 (GAS5‐wt) or GAS5 mutant vector (GAS5‐mut) with miR‐335 mimics or negative control (mimics NC). AC16 cells were harvested after transfected with miR‐335 mimics or mimics NC. D, Effect of GAS5 on the level of miR‐335. E, Effect of miR‐335 on the level of GAS5. Data are expressed as the mean ± standard deviation (SD), n = 3, **P < .01

As shown in Figure 4B, GAS5 transcript was found to contain a putative miR‐335 binding site. We found that GAS5 and miR‐335 were negatively co‐expressed in I/R‐heart tissues (Figure 4A). The luciferase assay was used to confirm whether GAS5 specifically binds to miR‐335. The result of luciferase assay indicated that luciferase activity in AC16 cells was significantly reduced by cotransfected with pmirGLO‐GAS5‐wt and miR‐335 mimics. While there is no significant change in luciferase activity when cotransfected with pmirGLO‐GAS5‐mut and miR‐335 mimics (Figure 4C). Furthermore, GAS5 knockdown was found to markedly increased miR‐335 expression, while miR‑335 inhibitor treatment significantly up‐regulate GAS5 expression in H9c2 and AC16 cardiomyocytes (Figure 4D and 4). To summary, these results suggested a reciprocal inhibition between miR‐335 and GAS5.

### 3.5 Down‐regulation of GAS5 inhibited ROCK1 via miR‐335

**Figure 5:**
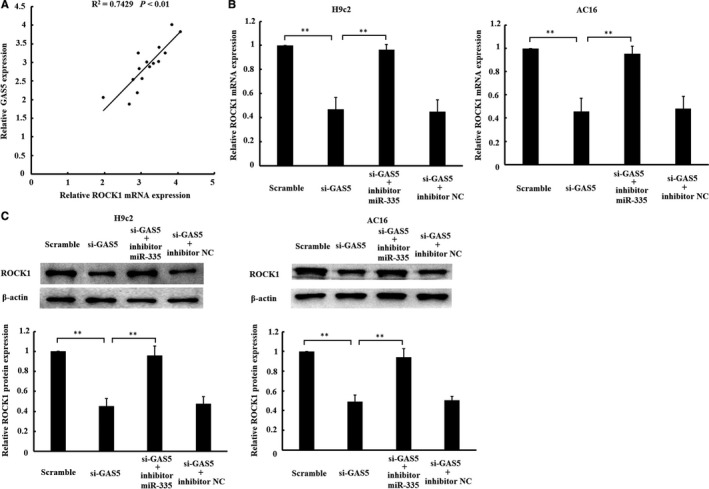
GAS5 regulates ROCK1 expression, and its effect is mediated by miR‐335. A, a positive relationship between GAS5 and ROCK1 expression existed in ischaemia/reperfusion (IR)‐heart tissues. Scramble and si‐GAS5 transfection alone or together with miR‐335 inhibitor or inhibitor NC was transfected into cardiomyocytes. 24 h after transfection, cells were subjected to 6 h of hypoxia, followed by 3 h of reoxygenation. B, mRNA and (C) protein expression levels of ROCK1 were detected by qRT‐PCR and Western blotting, respectively. Data are expressed as the mean ± standard deviation (SD), n = 3, **P < .01

Considering that ROCK1 is a known target of miR‐335^18^, ^19^ and GAS5 could regulate miR‐335 expression, we deduced that GAS5 might regulate ROCK1 mediated by miR‐335. As shown in Figure 5A, GAS5 and ROCK1 mRNA were positively co‐expressed in I/R‐myocardium. Moreover, the results of qRT‐PCR and Western blotting showed that mRNA and protein expressions of ROCK1 were down‐regulated by single transfection with GAS5‐siRNA in H/R‐cardiomyocytes, while the inhibition of the mRNA and protein expression of ROCK1 was abrogated by cotransfection with GAS5‑siRNA and miR‑335 inhibitor (Figure 5B and 5). These findings indicated that the regulation of GAS5 on ROCK1 was mediated by miR‐335.

### 3.6 Regulation of GAS5 on PTEN/PI3K/AKT/GSK‐3β/mPTP signal axis through miR‐335

**Figure 6:**
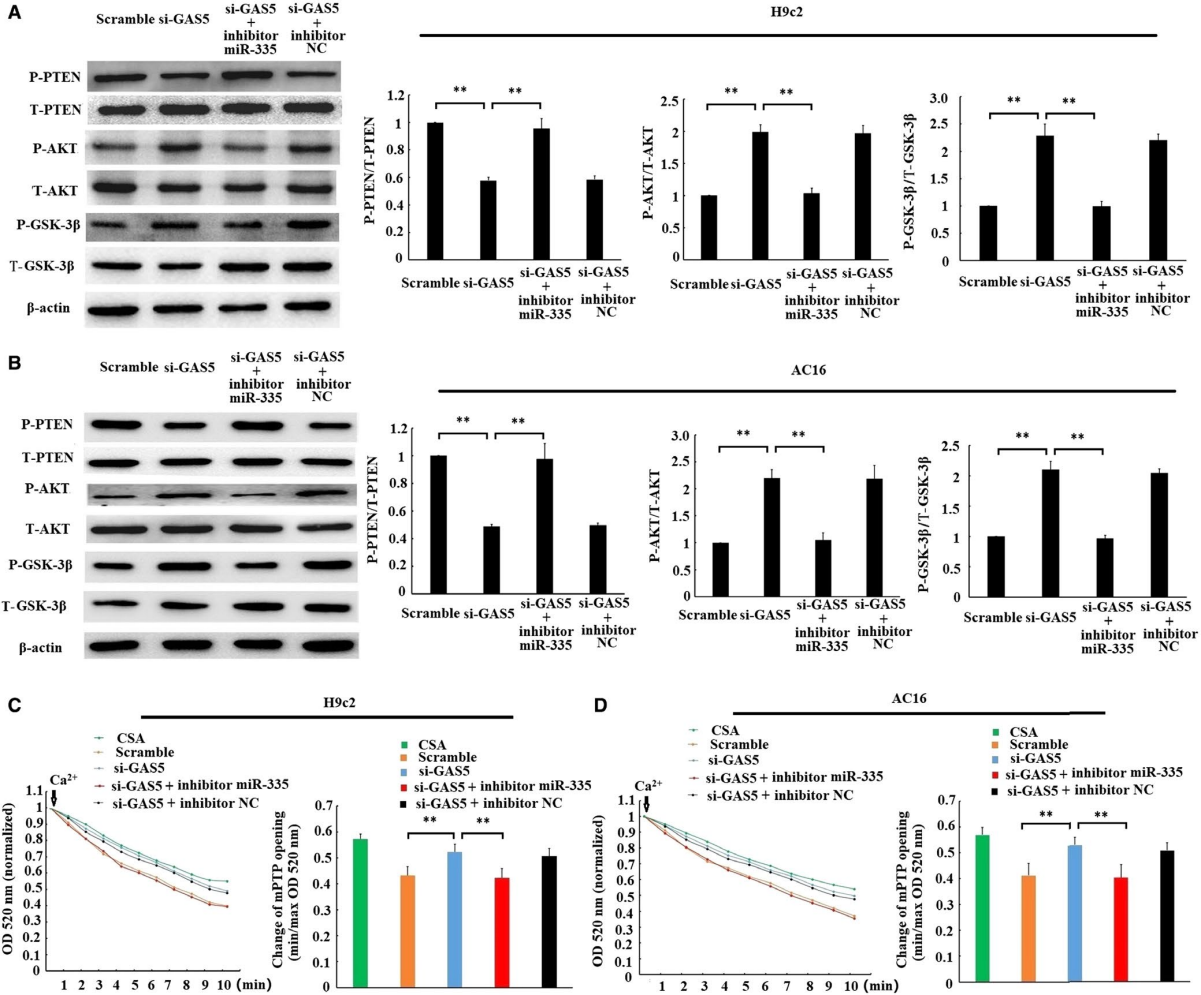
GAS5/miR‐335 manipulation regulated PTEN/PI3K‐AKT/GSK‐3β pathway and mitochondrial permeability transition pore (mPTP) opening. Scramble and si‐GAS5 transfection alone or together with miR‐335 inhibitor or inhibitor NC was transfected into cardiomyocytes. 24 h after transfection, cells were subjected to 6 h of hypoxia, followed by 3 h of reoxygenation. The phosphorylation levels of PTEN, AKT and GSK‐3β in H9c2 (A) and AC16 (B) cells were determined by Western blotting. The phosphorylation levels were expressed as the ratio of phospho‐protein expression to its corresponding total protein expression. mPTP opening was induced by CaCl_2_ in H9c2 (C) and AC16 (D) cells. The decrease of optical density (OD) value reflected the extent of mPTP opening. min OD, OD value recorded at the onset of experiment (0 min); max OD, OD value recorded at the end of experiment (10 min). min/max OD is negatively associated with the extent of mPTP opening. CSA, cardiomyocytes treated with 0.2 mmol/L cyclosporin a (CSA), a mPTP opening inhibitor, were used as a positive control. Data are expressed as the mean ± standard deviation (SD) n = 3, **P < .01

Several researches have demonstrated that the activation of ROCK1 could activate PTEN, further inactivate PI3K/AKT, subsequently leading in turn to GSK‐3β dephosphorylation and mPTP opening,^20^, ^21^ and we found that GAS5 could positively regulate ROCK1. Therefore, whether GAS5 regulates PTEN/PI3K/AKT/GSK‐3β/mPTP signal axis was further explored. As shown in Figure 6A and 6, the phosphorylation level of PTEN was decreased, while the phosphorylation level of AKT and GSK‐3β was enhanced by single transfection with the GAS5‐siRNA. However, cotransfection with the GAS5‑siRNA and miR‑335 inhibitor reversed the change in the phosphorylation level of PTEN, AKT and GSK‐3β. Similarly, the mPTP opening was inhibited by transfection with the GAS5‐siRNA alone in H/R‐cardiomyocytes, but the suppression was released by transfection with the GAS5‑siRNA and miR‑335 inhibitor together (Figure 6C and 6D). The results obtained suggested that GAS5 knockdown could inactivate PTEN, activate PI3K/AKT, subsequently leading in turn to inhibition of GSK‐3β and mPTP opening through regulation of miR‑335.

## 4 DISCUSSION

Growth arrest‐specific transcript 5 expression is commonly up‐regulated when exposed to lots of stressors, such as ischaemia,^8^, ^9^ CCl_4_ 22 and palmitic acid.^23^ In this study, we found that GAS5 expression was remarkably increased in cardiomyocytes and myocardium by H/R or I/R stimulation. This result was consistent with Liu SD et al finding^8^ in H/R‐cardiomyocyte and Hao S et al finding^9^ in MI mice. However, Li XX et al reported that GAS5 expression was significantly lower in the serum of patients with coronary artery disease (CAD) than that of healthy controls.^24^ This inconsistence may be attribute to the difference in the reaction to acute or chronic myocardial ischaemia.

GAS5 plays a regulatory role in pathophysiological process of liver fibrogenesis,^22^ tumorigenesis and metastasis,^25^ ischaemic stroke,^26^ etc Liu SD et al found that overexpression of GAS5 promoted myocardial apoptosis in H/R‐exposed cardiomyocyte,^8^ but whether down‐regulation of GAS5 alleviates myocardial I/R injury is not determined in their study. In our present study, we firstly demonstrated that down‐regulation of GAS5 in adult rat and human cardiomyocytes both prevented H/R‐induced injury, which confirmed that the function of GAS5 on myocardial I/R injury, is conserved. Furthermore, the cardioprotective effect by GAS5 knockdown on myocardial I/R injury was also reconfirmed in isolated rat hearts. Therefore, our findings suggested that GAS5 may be a new therapeutic target for the prevention of myocardial I/R injury in clinical practice. However, we cannot ignore the fact that the effect of GAS5 in other myocardial ischaemia models appears to be a discrepancy. Hao S et al suggested that up‐regulation of GAS5 attenuated AMI in mice.^9^ Similarly, Li XX et al implicated that up‐regulation of GAS5 alleviates myocardial injury in CAD rats.^24^ We speculated that this discrepancy may be ascribed to the difference in myocardial ischaemia model. Because pathophysiological process of myocardial I/R injury is different from simple myocardial ischaemia injury.^2^

GAS5 has been reported to interact with microRNAs through binding with each other.^22^, ^23^, ^26^, ^27^ For instance, GAS5 was found to bind to miR‐21 and the reciprocal negative regulatory relationships existed between miR‐21 and GAS5 in regulation of breast cancer.^27^ Similar regulatory relationships were also found between miR‐222 or miR‐26a and GAS5.^22^, ^23^ In our study, we identified that the specific binding and similar reciprocal inhibition also existed between miR‐335 and GAS5. These results were in line with Dai X et al finding^28^ and suggested a potential ceRNA regulatory network may exist between miR‐335 and GAS5.

ROCK, a major effector of small GTPase RhoA,^29^ is composed of two similar isoforms: ROCK1 and ROCK2.^30^ ROCK was activated at early myocardial reperfusion and may result in detrimental effects via inhibiting PI3K/AKT pathway.^31^ On the contrary, inhibition of ROCK activation prevented myocardial I/R injury through reactivating PI3K/AKT pathway.^32^ Considering that ROCK1 is a target of miR‐335,^18^, ^19^ we deduced that GAS5 acted a ceRNA for miR‐335 to regulate ROCK1. As expected, we found that inhibition of GAS5 repressed ROCK1 expression, subsequently activating PI3K/AKT pathway through regulation of miR‐335. This may partly account for the cardioprotection exerted by GAS5 knockdown.

Another thing worth noting is that although the disease models are distinct, the regulatory role of GAS5 on apoptosis is commonly reported, including our present study. Liu SD et al suggested that GAS5 could promote H/R‐induced apoptosis by up‐regulating LAS1 expression via p38/MAPK pathway.^8^ Hao S et al also reported that GAS5 could prevent AMI‐induced apoptosis through down‐regulating Semaphorin 3a.^9^ However, the mechanism for the regulation of apoptosis by GAS5 is not well‐illuminated. mPTP, a non‐specific pore located in the inner mitochondrial membrane, is an important regulator for apoptosis, especially mitochondrial‐mediated apoptosis. For instance, once mPTP opens, cytochrome *c* will be driven from the mitochondria into the cytoplasm, then caspases cascades are activated, and ultimately cell apoptosis will occur.^33^ GSK‐3β, a key upstream regulator of mPTP opening, is typically regulated by the PI3K/AKT‐mediated phosphorylation.^20^ mPTP opening will decrease, once GSK‐3β at Ser9 cite is phosphorylated.^34^ Our study demonstrated that GAS5 knockdown led to an increase in the phosphorylation level of GSK‐3β and repressed the mPTP opening through regulation of miR‐335. Therefore, our finding suggested that GAS5 regulated I/R‐induced apoptosis through GSK‐3β‐mediated mPTP opening and provided a new insight into the mechanism for GAS5‐regulated apoptosis.

However, there are two major limitations in this study. On the one hand, the effect of GAS5 on myocardial I/R injury was tested in an isolated rat heart model. The hearts in this model could not perfectly mimic the pathophysiological process of myocardial IR injury. Because it is deprived of neural and humoural regulation.^35^ Therefore, the regulatory role of GAS5 on myocardial I/R injury should be further confirmed in vivo. On the other hand, although we found that GAS5 knockdown protected against myocardial I/R injury, meanwhile it repressed ROCK1 and activated PI3K/AKT, whether activation of ROCK1 or inhibition of PI3K/AKT by genetic or pharmaceutical methods rescued the cardioprotection of GAS5 knockdown is still not confirmed. This is another limitation that we must acknowledge.

To summary, we demonstrate that down‐regulation of GAS5 prevents myocardial I/R injury may through regulating miR‐335/ROCK1/AKT/GSK‐3β axis. GAS5 may be a potential therapeutic target for myocardial I/R injury.

## CONFLICT OF INTEREST

The authors confirm that there are no conflicts of interest.

## AUTHOR CONTRIBUTIONS

Nan Wu, Dalin Jia and Chunyan Ma designed experiments. Nan Wu, Xiaowen Zhang, Yandong Bao and Hang Yu performed experiments. Xiaowen Zhang and Nan Wu wrote the manuscript. Nan Wu, Xiaowen Zhang, Chunyan Ma and Dalin Jia analysed data.

## Data Availability

The data sets analysed during the current study are available from the corresponding author on reasonable request.
